# Real-time imaging of senescence in tumors with DNA damage

**DOI:** 10.1038/s41598-019-38511-z

**Published:** 2019-02-14

**Authors:** Ying Wang, Jun Liu, Xiaowei Ma, Chao Cui, Philip R. Deenik, Paul K. P. Henderson, Ashton L. Sigler, Lina Cui

**Affiliations:** 1 Department of Chemistry and Chemical Biology, University of New Mexico, Albuquerque, NM 87131 USA; 2 UNM Comprehensive Cancer Center, University of New Mexico, Albuquerque, NM 87131 USA; 3 Departments of Biology and Biochemistry and Molecular Biology, University of New Mexico, Albuquerque, NM 87131 USA; 4 Present Address: Department of Medicinal Chemistry, College of Pharmacy, UF Health Science Center, UF Health Cancer Center, University of Florida, Gainesville, FL 32610 USA

## Abstract

Detection of cellular senescence is important not only in the study of senescence in various biological systems, but also in various practical applications such as image-guided surgical removal of senescent cells, as well as the monitoring of drug-responsiveness during cancer therapies. Due to the lack of suitable imaging probes for senescence detection, particularly in living subjects, we have developed an activatable near-infrared (NIR) molecular probe with far-red excitation, NIR emission, and high “turn-on” ratio upon senescence-associated β-galactosidase (SABG) activation. We present here the first successful demonstration of NIR imaging of DNA damage-induced senescence both *in vitro* and in human tumor xenograft models.

## Introduction

Senescence, a state of permanent cell-cycle arrest, plays an important role in tumor suppression, tumorigenesis and aging^1^. The hallmark of cellular senescence is growth arrest, primarily caused by the activation of cell-cycle inhibitors and tumor suppressors, therefore cells lacking senescence characteristics are cancer-prone^1^. DNA damaging agents such as chemotherapeutics can induce both cellular senescence and apoptosis, another cellular tumor-suppressive mechanism^2^. Of practical importance, DNA damage induced apoptosis – the primary target of anticancer therapy – has been widely accepted as an important factor in the determination of treatment outcomes for cancer patients^3^. Cellular senescence has been identified as an additional drug-responsive measure, particularly when many cell types become resistant to apoptosis in their senescent state^1,4^, rendering the detection of cellular senescence an urgent need.

Multiple agents are being developed for the detection of senescent cells, but most of these tools lack the capability of real-time imaging of senescence^5^. Due to the increased lysosomal biogenesis, cells at senescent state overexpress lysosomal beta-galactosidase (β-gal), and indeed senescence-associated β-gal (SABG) has been the most widely used biomarker for specific detection of senescent cells^6^. Many probes are available for the detection of β-gal, owing to the widespread utility of reporter gene *LacZ*, a bacterial gene that encodes β-gal. The routinely used substrate X-Gal (5-bromo-4-chloro-3-indolyl-β-D-galactoside) yields a blue insoluble indigoid dye in the presence of β-gal, making it suitable for β-gal detection via histochemical staining *in vitro*^7^. Pioneered by Nagano and Urano, a series of fluorescein and rhodamine derived probes have been developed for β-gal detection in *LacZ*(+) live cells^8–11^. Near infrared (NIR) fluorescence is favored for *in vivo* studies due to the decreased tissue autofluorescence, high penetration depth, and low light scattering^12^. Weissleder and colleagues developed a far red fluorescence probe DDAOG, a β-galactoside of 7-hydroxy-9H-(1,3-dichloro-9,9-dimethylacridin-2-one), for the detection of *LacZ*(+) model tumor *in vivo*^13^. A bioluminescent probe, a conjugate of β-galactoside and luciferin, allowed detection of β-gal in a mice model injected with engineered cell line expressing both *LacZ* and *fLuc* (encoding firefly luciferase)^14^. Via photoacoustic imaging, Wang and co-workers were able to detect β-gal activity in *LacZ*(+) cells *in vivo* using X-Gal as substrate^15^. Recently, a couple of groups showed the detection of endogenous β-gal in several rare cases of cancers. For example, Urano and colleagues applied their fluorogenic probe hydroxymethyl rhodol (HMR) β-galactoside, with 1,400-fold fluorescence turn-on ratio, for visualization of small peritoneal metastatic tumors^16^. A ratiometric near-infrared fluorescent probe was developed for real-time tracking and imaging of β-gal activity in colorectal tumor *in vivo*^17^. While all of the previously reported probes for β-gal could be possibly useful for senescence detection, their applicability remains to be evaluated. Recently, a two-photon fluorescence probe for senescence was reported, however the emission wavelength at 540 nm limits its utility for deep tissue imaging^18^.

We have developed an activatable NIR molecular probe with far-red excitation and NIR emission (708 nm), and a 130-fold enhanced fluorescence intensity upon β-gal activation. We present here the first demonstration of NIR imaging of DNA damage-induced senescence in xenograft human tumors in living mice.

## Results and Discussion

### Design, synthesis and activation of the NIR probe by recombinant β-gal

**Figure 1:**
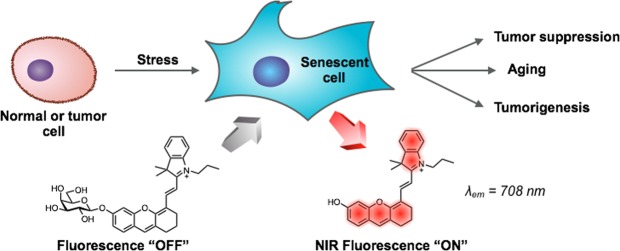
Fluorescence detection of cellular senescence using the NIR-BG probe. *λ*_ex_ = 680 nm; *λ*_em_ = 708 nm.

The fluorescence of the probe is designed to “turn-on” in the presence of senescence-associated β-gal (SABG) – the probe that remains non-fluorescent without SABG fluoresces when SABG is expressed (Fig. 1). A hemicyanine derivative (Fig. 1) was selected as the fluorophore for its excellent stability, low toxicity, and fluorescence emission in NIR range upon activation^19^. The fluorophore has demonstrated its suitability for fluorescence imaging of various biomolecules, such as the detection of β-Lactamase in *Staphylococcus aureus*^20^, the detection of nitroxyl (HNO)^21^ and palladium^22^ in live cells, the detection of nitroreductase^23^, γ-glutamyl transpeptidase^24^ and tyrosinase^25^ in zebrafish. More recently, the hemicyanine NIR dyes have been used for *in vivo* detection of cysteine^26,27^, alkaline phosphatase in tumor models^28–30^, superoxide radical anion^31^, hydrogen sulphide^32^, hydrogen polysulfides^33^ and γ-glutamyl transpeptidase^34^ in mice models.

Therefore, the probe NIR-BG (NIR probe for β-gal) was efficiently synthesized by glycosylation of the hemicyanine dye and a protected galactosyl bromide, followed by a deprotection step (Figs S3–10). The fluorescence of the NIR-BG was initially at its background due to the reduced electron-donating strength of the oxygen atom upon glycosylation; upon cleavage of the galactose, the fluorescence of the hemicyanine restored (turned-on) by regaining its zwitterionic resonance form^19^.

**Figure 2:**
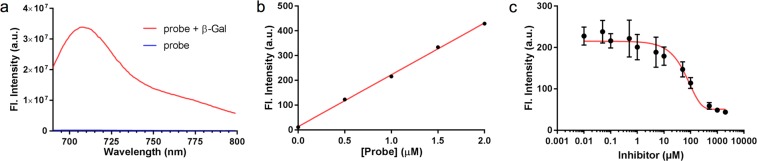
Activation of the NIR-BG probe by recombinant β-gal. (**a**) Fluorescence spectra of probe (blue line) and probe (10 μM) with 2 unit (U) of β-gal (red line); *λ*_ex_ = 680 nm; t = 5 minutes; T = 37 °C. (**b**) Activation of probes at various concentrations (0, 0.5, 1.0, 1.5, 2.0 μM) by β-gal (2 U); t = 5 minutes; T = 37 °C; *R*^2^ = 0.9988. (**c**) Inhibition study of β-gal using IPTG and NIR probe (5 μM).

We then evaluated the “turn-on” efficiency of the NIR-BG probe using recombinant β-gal from *E. coli*. The intensity of the fluorescence emission of the probe with and without the enzyme was compared (Figs 2a, S1). While the fluorescence of the probe in PBS buffer remained at baseline intensity, the reaction of the probe with β-gal produced a strikingly more intense fluorescence signal peaked at 708 nm (NIR range), with a fluorescence on/off ratio of 130-fold within 5 min (Fig. 2a). The fluorescent product had identical optical properties as the uncaged hemicyanine dye. With constant amount of β-gal, the fluorescence intensity is linearly proportional to the probe concentration, with a linear correlation coefficient of determination *R*^2^ of 0.9988 (Fig. 2b). High-performance liquid chromatography (HPLC) analysis was conducted to confirm the enzymatic hydrolysis process (Fig. S2). Our probe itself eluted at 11.87 min; when the probe was exposed to β-gal, a new peak at 11.65 min appeared, which was attributed to the enzymatic hydrolysis product, based on the same retention time, UV-vis absorption, and mass spectrometry as the standard hydrolyzed/uncaged hemicyanine dye. This result confirmed that our probe was efficiently activated by β-gal. Also, as shown in the Fig. 2c, the fluorescence intensity dropped with the increase of the β-gal inhibitor (IPTG, isopropyl thio-β-D-galactoside) concentration, further confirming the probe activation is through galactose hydrolysis by β-gal, and our probe may work as a tool for β-gal inhibitor screening. The stability assay showed the probe is stable with 30 times of laser excitation or 48 hours incubation with β-gal (Fig. S11).

### Kinetics and detection limit of β-gal by the NIR-BG probe

**Figure 3:**
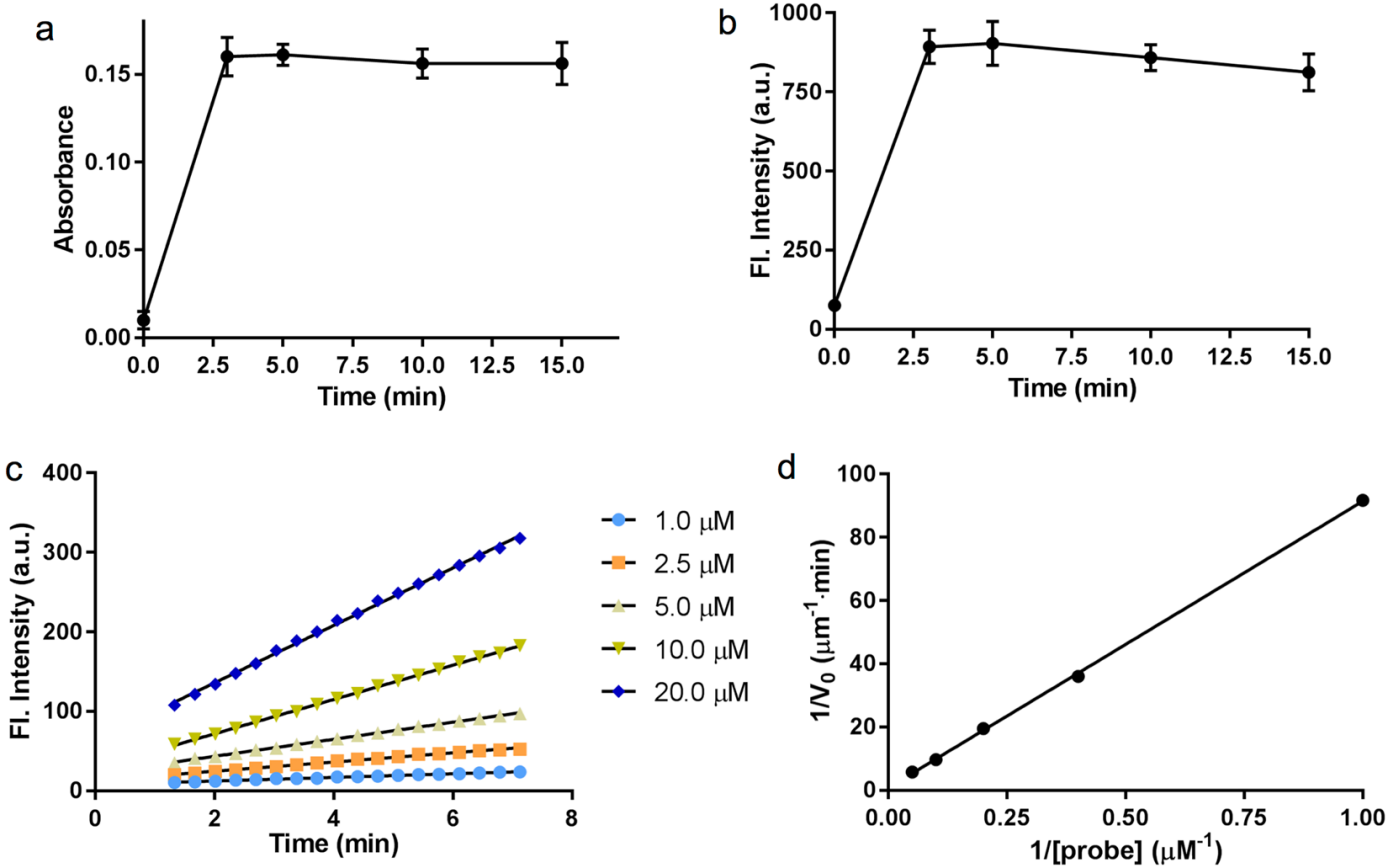
Kinetics of the NIR-BG probe activation by β-gal. (**a**) Exitation and (**b**) Emission of 5 μM NIR probe in the presence of β-gal (10 U/mL), *λ*_ex_ = 680 nm, *λ*_em_ = 700 nm. (**c**) Time-dependent fluorescence intensity increment at 700 nm using β-gal (0.1 U/mL) with different amounts of probes. *λ*_ex_ = 680 nm; *λ*_em_ = 700 nm. (**d**) Lineweaver-Burke plot of the probe activation by β-gal.

With excess amount of enzyme, the NIR-BG probe could be fully activated within 3 minutes (Fig. 3a,b). To quantify the kinetic parameters, time-dependent fluorescence intensity was measured in the presence of β-gal and probes at different concentrations (Fig. 3c). A good linear equation was obtained at various probe concentrations: 1 μM, R^2^ = 0.9943; 2.5 μM, R^2^ = 0.9939; 5 μM, R^2^ = 0.9988; 10 μM, R^2^ = 0.9994; 20 μM, R^2^ = 0.9984. The reaction rate was found to increase with probe concentrations (Fig. 3d). The Lineweaver-Burke plot of the probe hydrolysis by β-gal was generated based on the slope of each line in Fig. 3C. The reaction rate was calculated using Lineweaver-Burk equation, 1/*V*_0_ = *K*_*M*_/(*K*_*cat*_[*E*_0_][*S*]) + 1/(*K*_*cat*_[*E*_0_]), where [*E*_0_] is the concentration of β-gal. The kinetic parameters *K*_*M*_, *K*_*cat*_ and *K*_*cat*_/*K*_*M*,_ were calculated to be 106 μM, 3.3 s^−1^, and 0.31 μM^−1^•s^−1^, respectively. The Michaelis constant (*K*_*M*_) of our probe was much lower than that of commercial X-Gal. The value of *K*_*cat*_/*K*_*M*_ of probe was 16-fold higher than that of X-Gal (*K*_*cat*_/*K*_*M*_ = 8.29 × 10^−3^ μM^−1^•s^−1^), suggesting the high activation efficiency of our probe by β-gal.

**Figure 4:**
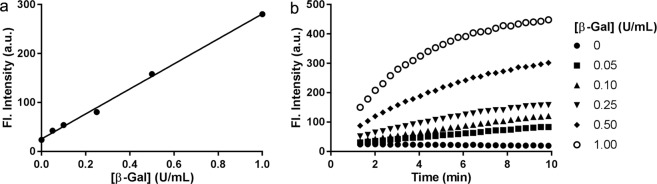
Detection limit of β-gal by the NIR-BG probe. (**a**) Probe activation by various amounts of β-gal in 3 min. (**b**) Probe activation by various amounts of β-gal over time. Probe (5 μM), *λ*_ex_ = 680 nm, *λ*_em_ = 700 nm.

In order to evaluate the detection limit of our NIR-BG probe for β-gal, the probe was incubated with β-gal at various concentrations (Fig. 4). Within 3 min, the probe fluorescence could be detected with a good linear correlation (R^2^ = 0.9972). Based on 3σ/s method (see Supporting Information)^35^. The limit of detection (LOD) was calculated to be 0.0031 unit/mL (0.13 nM), indicating the high sensitivity of our probe^36^.

### Detection of β-gal in *LacZ*(+) live cells

**Figure 5:**
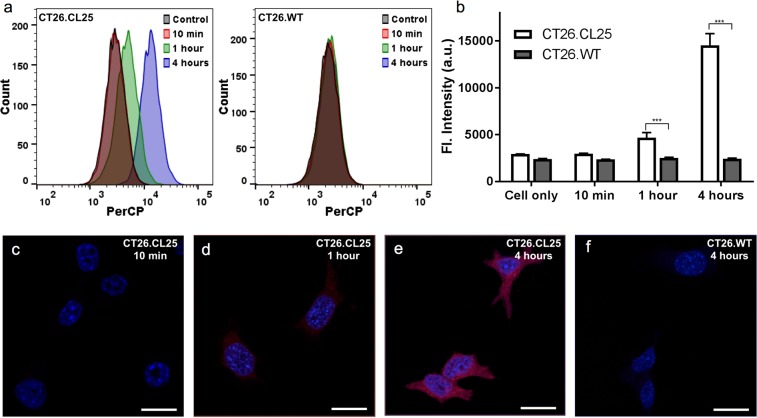
Detection of β-gal by the NIR-BG probe in *LacZ*(+) cells (CT26.CL25). (**a**) Flow cytometry of CT26.CL25 (left) and negative control CT26.WT cells (right); probe (10 μM), *λ*_ex_ = 640 nm. (**b**) Quantification of the mean fluorescence intensity in (**a**). (**c**–**f**) Confocal fluorescence microscopy images of the NIR probe (10 μM) incubated with CT26.CL25 cells for 10 min (**c**), 1 hour (**d**) and 4 hours (**e**), and CT26.WT cells for 4 hours (**f**). Blue, DAPI; red, probe. ***P < 0.001.

In order to see whether our NIR-BG probe can be used for β-gal detection in live cells, we chose an engineered cell line that stably express *LacZ*(+), the gene that encodes β-gal. CT26.CL25, a mouse colon fibroblast carcinoma cell line that express β-gal, was therefore used in our initial live cell experiments. CT26.WT, the wild type cell line that does not express β-gal, was used as a control. Our probe did not appear toxic in neither cell culture after 24-hour incubation (Fig. S12). Cell samples after incubating with our NIR-BG probe were analyzed using flow cytometry (Fig. 5a,b) and confocal microscopy (Fig. 5c–f). Flow cytometry showed obvious fluorescence at 1 hour and 4 hours in CT26.CL25 cells incubated with our probe, whereas only background signal was detected in CT26.WT cells that received the same treatment (Fig. 5a,b). Confocal fluorescence microscopy images showed consistent results. Fluorescence signal was obvious in samples with 1-hour probe treatment, and the intensity increased with time in CT26.CL25 cells (Fig. 5c–e); while no fluorescence in the NIR channel was detected in the CT26.WT cells even after 4-hour incubation (Fig. 5f). The NIR signal in CT26.CL25 cells were highly colocalized with the β-gal expression (Fig. S13). Clearly, the fluorescence detected in CT26.CL25 cells using both flow cytometry and confocal microscopy was from our NIR-BG probe after hydrolysis of the galactose by β-gal, while no fluorescence was detected in CT26.WT due to the lack of β-gal. The β-gal expression was confirmed by colorimetric X-gal staining. Intensive blue stain was observed in CT26.CL25 cells, indicating high β-gal expression compared with the wild type cell line (Fig. S14A).

### Imaging of senescent state in drug or radiation treated human cancer cells

**Figure 6:**
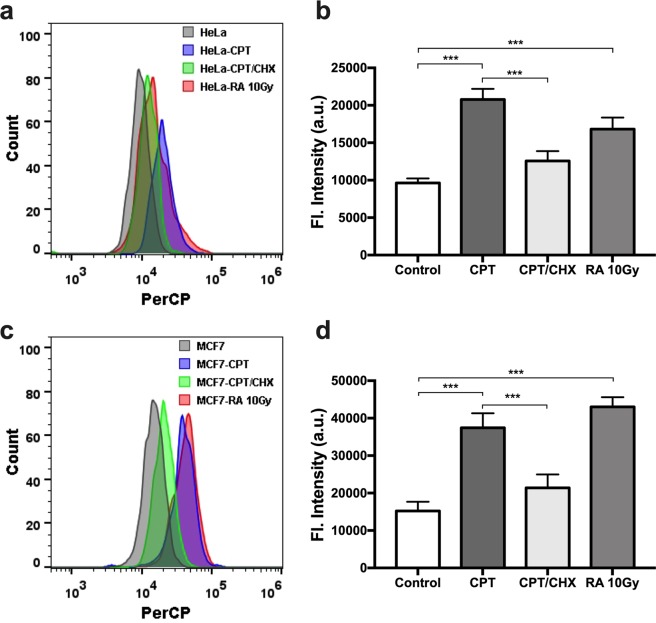
Detection of senescence in human cancer cells via flow cytometry. Flow cytometry (**a**) and quantification (**b**) of HeLa cells without treatment, or with CPT, CPT/CHX, or radiation treatment. Flow cytometry (**c**) and quantification (**d**) of MCF7 cells without treatment, or with CPT, CPT/CHX, or radiation treatment. Probe concentration, 10 μM. *λ*_ex_ = 640 nm; PerCP bandpass filter at 675/25 nm. ***P < 0.001.

With the confidence of our probe being able to detect β-gal in live cells, we moved further to evaluate whether it could differentiate senescent cells and normal cells. Human cervical cancer cells (HeLa) and metastatic breast cancer cells (MCF7), were used in all four groups of cells evaluated. Senescence in HeLa and MCF7 cells were induced using camptothecin (CPT), an inhibitor of DNA topoisomerase I that could cause lethal DNA strand breaks^37^. Interestingly, when the cells were incubated with both CPT and cycloheximide (CHX), an experimental blocker of protein synthesis, cellular senescence could be alleviated^38^. Radiation therapy is another effective method for cancer treatment in clinic; it can also induce cell cycle arrest leading to senescence. All four groups of cells, with no drug treatment, with CPT treatment, with both CPT and CHX treatment, or with 10 Gy radiation treatment, were then incubated with our NIR-BG probe, followed by flow cytometry (Fig. 6). Importantly, CPT or radiation treated cells had significantly higher fluorescence compared to the untreated control cells; the higher fluorescence presumably resulted from the expressed senescence-associated β-gal (SABG), which turned on the fluorescence of the NIR-BG probe. Meanwhile, the fluorescence in cells treated with both CPT and CHX was significantly reduced comparing to the CPT-treated group, suggesting the suppressed senescence due to CHX treatment. Same trend was observed for both HeLa and MCF7 cells (Fig. 6).

**Figure 7:**
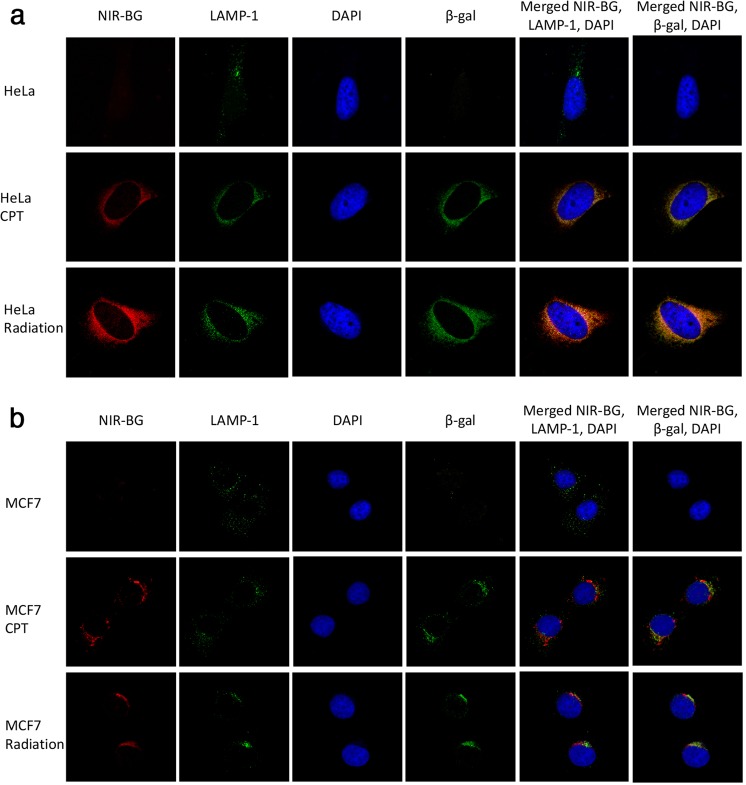
Confocal microscopy of senescent cells. (**a**) HeLa cells and (**b**) MCF7 cells treated with PBS, CPT, or radiation, followed by incubation with NIR-BG probe (10 μM) for 1 h. Signal: blue, DAPI; red, NIR-BG probe; green, lysosome or β-gal.

Since SABG is localized in the lysosome, we then performed confocal microscopy of the normal and senescent HeLa or MCF7 cells (Fig. 7). Fluorescence from the NIR-BG probe (NIR-BG channel) in CPT or radiation treated cells was strikingly higher than that of the control cells without treatment. More importantly, the red fluorescence from our probe overlapped mainly with the lysosomes (LAMP-1 channel), suggesting the high probability that our probe was activated in lysosomes, where SABG was expressed. This was further confirmed by co-staining the cells with anti-β-gal antibody (β-gal channel); our probe signal in senescent cells, induced either by chemotherapeutics or by radiation, was well colocalized with both lysosome and β-gal (Fig. 7). This feature was observed in both HeLa cell groups and MCF7 cell groups. These results further confirmed that the NIR-BG could image chemotherapy and radiation therapy induced senescence in different types of cancer cells. X-gal staining also verified the existence of SABG in cells after chemotherapy and radiation therapy (Fig. S14B).

**Figure 8:**
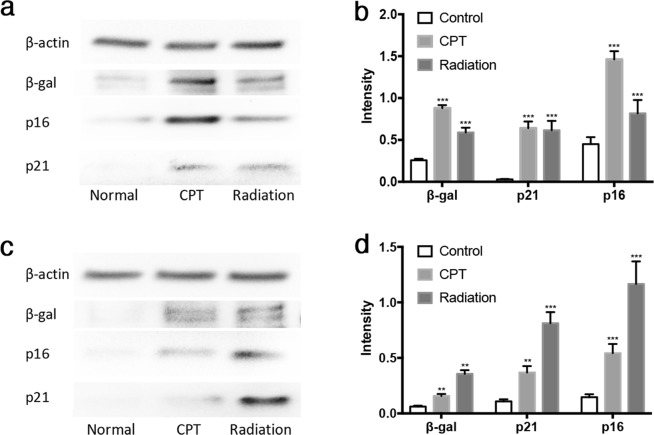
Western blot of cell lysates. (**a**) Western blot and (**b**) quantification of β-gal, p16, and p21 expression in HeLa cells without drug treatment, with CPT or radiation treatment. (**c**) Western blot and (**d**) quantification of β-gal, p16, and p21 expression in MCF7 cells without drug treatment, with CPT or radiation treatment. ***P < 0.001, **P < 0.005.

Cell cycle inhibitors p21 and p16 are often expressed by senescent cells^1^. Therefore, we examined the cell lysates of all cell samples from the imaging studies using Western blot (Figs 8 and S15). Western blot indicated that tumor suppressors p16 and p21 both increased in HeLa cells treated with CPT or radiation compared with normal HeLa cells (Fig. 8a,b), confirming the cell samples we used were indeed senescent, and the induction of senescence by CPT or radiation was at least partially through p53 pathway, as p21 is typically induced directly by p53. In addition to HeLa cells, MCF7 cells also expressed higher levels of p16 and p21 upon CPT treatment (Fig. 8c,d). The Western blot results confirmed the senescent state of these drug or radiation treated cells used in the imaging experiments. Interestingly, β-gal levels in drug or radiation treated HeLa or MCF7 cells were all elevated compared with the control cells, further suggesting the activation of β-gal enzymatic activity during senescence (Fig. 8).

### Real-time imaging of senescence in tumor models

Successful detection of cellular senescence *in vitro* allowed us to further examine the capability of NIR-BG to visualize senescence in tumors in living mice. It is important to note that unactivated NIR-BG’s absorption peaks around 640 nm and emission peaks around 660 nm, while the activated probe NIR-BG has the maximal absorption and emission at 680 nm and 710 nm respectively (Fig. S1). The imaging instrument IVIS spectrum can take advantage of this major difference between the unactivated and activated probe, therefore we examined our animals using two different filter settings (Ex640 nm/Em680 nm for unactivated probe and Ex675 nm/Em720 nm for the activated probe).

**Figure 9:**
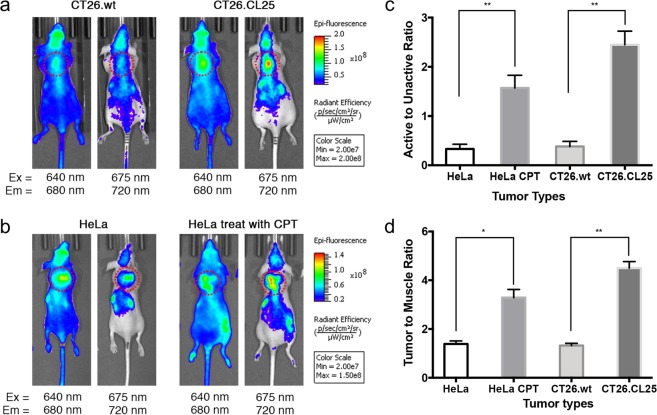
Fluorescence imaging of mice bearing CT26 tumors (**a**), HeLa tumors with or without CPT treatment (**b**), quantification of the probe activation (**c**), and tumor-to-muscle ratio (**d**). Red dotted cycle: tumor position.

In a preliminary experiment, we used the genetically modified mice colon cancer cell line CT26 to determine whether NIR-BG could differentiate tumors with and without active β-gal (Fig. 9a). The *LacZ*(+) CT26.CL25 tumors showed significantly higher signals of NIR-BG than CT26.WT tumors at 1 h after probe injection using 675 nm excitation and 720 nm emission filter setting; while their difference was minimal when using 640 nm excitation and 680 nm emission setting. This result suggested the unactivated form of NIR-BG could distribute to tumor with little influence by the β-gal expression, and the probe could be activated in β-gal expressing tumors therefore was detected upon 675 nm excitation and 720 nm emission. We also confirmed β-gal expression in resected whole tumor and tumor slides (Fig. S17). Immunofluorescent staining of the resected tumor tissues also showed homogenous distribution of probe NIR-BG which overlapped with β-gal in the CT26.CL25, but not in the CT26.WT tumors (Fig. S18).

We then moved on to HeLa xenografted mice models to see whether probe NIR-BG could visualize chemotherapy-induced senescence in tumors. Tumor-bearing mice were first systemically treated with CPT or saline before the probe NIR-BG was injected. With the treatment of CPT, the tumor growth was significantly inhibited compared to the mice group receiving saline only (Fig. S16). We were delighted to find that NIR-BG behaved similarly in the drug-treated mice (with drug-induction triggered expression of β-gal) to the CT26 tumor models (with genetically expressed β-gal) (Fig. 9). HeLa tumors receiving either CPT treatment or saline showed similar signal of NIR-BG while using the 640 nm excitation and 680 nm emission filter setting, however, there was a significantly higher signal from the activated form of NIR-BG (observed using 675 nm excitation and 720 nm emission filter) in the HeLa tumors with CPT treatment (Fig. 9b). The result was also quantified in Fig. 9. Resected intact whole tumor staining, histochemical staining with X-gal (Fig. S17) and immunofluorescent staining of tumor tissue with antibodies against β-gal (Fig. S19) further confirmed the probe activation in senescent tumors.

## Conclusion

The importance of detecting cellular senescence lies not only in biological studies of senescence in various biological systems, but in various practical applications such as image-guided surgical removal of senescent cell, as well as the monitoring of drug-responsiveness during cancer therapies. Due to the lack of suitable imaging probes for senescence detection, particularly in living subjects, we have developed an activatable NIR-BG molecular probe with far-red excitation, NIR emission, and high “turn-on” ratio upon β-gal activation. We present here the first demonstration of NIR imaging of DNA-damage induced senescence in xenograft human tumor models. In the *in vitro* work, we observed fluorescence in cells with knocked-in *LacZ* as well as senescent cells induced by drug or radiation treatment. The fluorescence signal co-localized with lysosomes in senescent cells, suggesting the presence of SABG in lysosome, one of the key features in cellular senescence. Cell cycle inhibitors p16 and p21 had elevated expression in cells with enhanced fluorescence signal, confirming the induction of cellular senescence in the cell studies. We finally examined our probe in mice bearing either *LacZ*(+) tumor or drug-treated xenograft human tumor models, and the probe NIR-BG was able to detect both genetically expressed β-gal or drug-induced SABG, suggesting the potential applicability of using NIR-BG to evaluate the efficacy of cancer therapeutics.

## Materials and Methods

### Materials

Our probe was prepared following a literature procedure (also see Supporting Information)^23,39^. Other chemical reagents and MTT (3-(4,5-dimethylthiazol-2-yl)-2,5-diphenyltetrazolium bromide) were purchased from Sigma-Aldrich and Alfa Aesar. DAPI was from Biotium, CA, USA. Bovine serum albumin and PBS were obtained from Amresco, OH, USA. DMEM and RPMI-1640 were from Corning Inc, USA. β-Galactosidase (*E. coli*) was purchased from Abnova. Fetal Bovine Serum (FBS) was from VWR. Anti-LAMP1-lysosome antibody (Catalog #ab79821), anti-β-galactosidase antibody (Catalog #ab9361), goat anti-rabbit IgG H&L-AF488 (Catalog #ab150081), goat anti-chicken IgY H&L-AF568 (Catalog #ab175477), anti-EEA1 antibody (anti-Endosome antibody), anti-p16 antibody (Catalog #108349), and anti-p21antibody (Catalog #109520) were from Abcam (USA). RIPA cell lysis buffer was from Enzo Life Sciences, NY, USA. ProLong Gold Antifade Mountant was from Invitrogen, USA. HeLa (human cervical cancer cell line), MCF7 (human breast cancer cell line), CT26.CL25 and CT26.WT (mouse colon fibroblast carcinoma) cell lines were purchased from American Type Culture Collection (ATCC), VA, USA.

### *In vitro* enzymatic assay

Probe was used at a final concentration of 5 μM. Absorption and fluorescence spectra of probe with 2-unit β-gal enzymatic reactions were performed at 37 °C in a 200 μL total volume of PBS buffer for 3 min, 5 min, 10 min and 15 min. In addition, fluorescence intensity of 2 μM probe was performed with 0.25, 0.5, 1, 2, 4 units of β-gal for 5 mins.

### Cells and culture conditions

HeLa and MCF7 cells were cultured at 37 °C in 10 cm dishes containing Dulbecco’s Modified Eagle’s medium (DMEM) supplemented with 10% fetal bovine serum, and antibiotics (100 U/mL penicillin, 100 μg/mL streptomycin) under 5% CO_2_ and 95% humidity. CT26.CL25 and CT26.WT cells were cultured in complete RPMI-1640 medium supplemented with 10% FBS and antibiotics (100 U/mL penicillin, 100 μg/mL streptomycin) at 37 °C with 5% CO_2_ and 95% humidity.

### Induction of cellular senescence

HeLa and MCF7 cells were seeded in a 24-well plate at a density of 2 × 10^3^ cells/well. Then cells were cultured in the presence of 7.5 nM camptothecin (CPT) or CPT together with 0.15 μM cycloheximide (CHX) for a week. And for radiation therapy induced cellular senescence, cells were irradiated with 10 Gy X-ray (2 Gy/min) and then were cultured for another 48 hours.

### Immunofluorescence cell staining

Cells were seeded in a 24-well plate coated with cover slides at a density of 3 × 10^4^ cells/well. Then cells were incubated with 10 μM probe for 10 min, 1 hour and 4 hours, and fixed with 4% paraformaldehyde for 30 min. Cells were blocked with 5% bovine serum albumin (BSA) followed by washing three times with PBS and incubated with anti-LAMP1-lysosome antibody (1:100) and anti-β-galactosidase antibody (1:500) at 4 °C overnight. In the next day, cells were subsequently washed three times with PBS, stained with goat anti-rabbit IgG H&L-AF488 (1:200) and goat anti-chicken IgY H&L-AF568 (1:500) at room temperature for 1 hour, and counter-stained with diamidino-phenyl-indole (DAPI, Catalog #40011, Biotium, USA) for 5 min. After washing, all cover slides were mounted by ProLong Gold Antifade Mountant (Catalog #P36934, Invitrogen, USA), and images were acquired using a confocal laser scanning microscopy (Leica TCS SP8, Germany).

### X-gal staining

Cells were seeded in a 24-well plate coated with cover slides at a density of 3 × 10^4^ cells/well. After inducing senescence, cells were fixed with 4% paraformaldehyde for 5 min and then stained with X-gal staining solution (40 mM citric acid/sodium phosphate buffer, 5 mM K_4_[Fe(CN)_6_] 3H_2_O, 5 mM K_3_[Fe(CN)_6_], 150 mM sodium chloride, 2 mM magnesium chloride, and 1 mg/ml X-gal) for 6 hours at 37 °C. The cover slides were mounted by ProLong Gold Antifade Mountant and were scanned using Olympus DSU-MBF Stereology System (Olympus, Japan).

### Cell viability assays

Cell growth and cytotoxicity were evaluated by MTT (3-(4,5-dimethylthiazol-2-yl)-2,5-diphenyltetrazolium bromide) assay. Cells were seeded in a 96-well plate at a density of 1 × 10^4^ cells/well, then cells were incubated with 0, 0.5, 1, 2, 5, 10 and 20 μM probe for 4 hours and 24 hours at 37 °C. After removal of the old medium, MTT reagent (3 mg/mL) was added, and cells were incubated for 3 hours at 37 °C. Upon the formation of visible purple crystals (formazan), medium was aspirated and dimethyl sulfoxide (DMSO, 100 μL) was added to dissolve the formazan for 20 min. Absorbance was read at 570 nm and cell viability was calculated based on untreated wells.

### Flow cytometry

Cells were seeded in a 24-well plate at a density of 3 × 10^5^ cells/well with or without treatment. Then cells were incubated with 10 μM of probes for 10 min, 1 hour and 4 hours at 37 °C. Cells were then washed with PBS buffer and digested with 0.25% trypsin. Cells were collected into Eppendorf tubes, washed with PBS buffer 3 times at 300 × *g* for 4 min, and resuspended in 50 μL PBS buffer. The fluorescence of cell samples (3 samples for each data point) was analyzed with a flow cytometer (Accuri C6 Plus, Becton Dickinson and Company, USA).

### Western blot analysis

Proteins from both treated and untreated cells were extracted with radioimmunoprecipitation assay (RIPA) buffer. Total protein was quantifed using the Pierce™ BCA (bicinchoninic acid) Protein Assay Kit (ThermoFisher Scientific). Samples (25 μg) were separated on 10% SDS-PAGE (sodium dodecyl sulfate polyacrylamide gel electrophoresis) gels and transferred to PVDF (polyvinylidene fluoride) membranes. After being incubated in 5% skim milk, the PVDF membranes were incubated with the primary antibody at 4 °C overnight and then with the secondary antibody for 1 hour at room temperature. Immunodetection was performed using enhanced chemiluminescence (ECL), and the protein bands were quantified using ImageJ software. Relative protein levels were calculated by normalization to β-actin.

### *In vivo* animal imaging

*In vivo* imaging was performed in accordance with protocols approved by the Institutional Animal Care and Use Committee of the University of New Mexico following the National Institutes of Health guidelines for animal care. 2 × 10^6^ HeLa cells or 5 × 10^4^ CT26.WT or CT26.CL25 cells were injected subcutaneously to athymic female nude mice (Harland Laboratories) to establish tumor models. For the CT26.WT and CT26.CL25 tumor models, NIR-BG (10 nmol) was injected through tail vein when tumor size reached 150 mm^3^. The animals were then subjected to fluorescence imaging at 1 h post injection. For the HeLa xenografted mice, when tumor size reached 100 mm^3^, one group of the mice (5 mice per group) were treated with CPT (2 mg/kg every two days for 4 times, total dosage at 8 mg/kg) while the control group (5 mice per group) was treated with the same volume of saline. Two days after the last treatment, mice were injected with the NIR-BG probe (10 nmol) for fluorescence imaging at 1 h post injection. Animal imaging was performed using an IVIS spectrum (PerkinElmer, USA) optical imaging system with two filter settings (Ex640 nm/Em680 nm for unactivated probe and Ex750 nm/Em720 nm for activated probe). After live animal imaging, the mice were euthanized and tumors were resected for immunohistochemistry staining and imaging.

### Immunohistochemistry staining

The tumors were immersed in optimal cutting temperature (OCT) compound and were frozen on dry ice immediately after resection. The tissues were then sectioned into 4 μm slides and were fixed with 4% paraformaldehyde for 5 min. The slides were then counter-stained with anti-β-gal antibody (1:250) and anti-LAMP1-lysosome antibody (1:250) for immunofluorescent histology imaging and counter-stained with eosin and X-gal (for β-gal).

### Statistical analysis

Values are reported as the mean ± standard deviation unless otherwise noted. Student’s t-test and two-way analysis of variance (ANOVA) were used to determine the statistical significance with probability values less than 0.05. All statistical calculations were performed using Prism 7.0 (GraphPad Software).

## Supplementary information

Real-time imaging of senescence in tumors with DNA damage
