# Supplementary material for: Real-time imaging of senescence in tumors with DNA damage
Source: Sci Rep. 2019 Feb 14;9:2102. doi: 10.1038/s41598-019-38511-z (PMC6375927; doi:10.1038/s41598-019-38511-z)
Supplement: Supplementary file 1 — Real-time imaging of senescence in tumors with DNA damage [file 41598_2019_38511_MOESM1_ESM.docx]

**Real-time imaging of senescence in tumors with DNA damage**

Ying Wang ^1, 2,^ *^#^*, Jun Liu ^1, 2,^ *^#,^*^&^, Xiaowei Ma^1, 2,^ *^#,^*^&^, Chao Cui^1, 2,^*^,^*^&^, Philip R. Deenik ^1, 2^, Paul K.P. Henderson ^1, 2^, Ashton L. Sigler ^1,2,3^, and Lina Cui ^1, 2, &,*^

***^1^*** Department of Chemistry and Chemical Biology, University of New Mexico, Albuquerque, NM 87131, USA.

***^2^*** UNM Comprehensive Cancer Center, University of New Mexico, Albuquerque, NM 87131, USA.

***^3^*** Departments of Biology and Biochemistry and Molecular Biology, University of New Mexico, Albuquerque, NM 87131, USA.

***^#^***These authors contributed equally to this work.

^&^Current Address: Department of Medicinal Chemistry, College of Pharmacy, UF Health Science Center, UF Health Cancer Center, University of Florida, Gainesville, FL 32610, USA

*Correspondence and requests for materials should be addressed to L.C. (email: [linacui@cop.ufl.edu](mailto:linacui@cop.ufl.edu)).

**Supplementary information**

**General methods**

Deuterated solvents were purchased from Sigma-Aldrich and Merck Millipore. Compound 1[[1](#_ENREF_1)] and 2[[2](#_ENREF_2)] were prepared following a literature procedure. ^1^H and ^13^C NMR spectra were recorded on a Bruker instrument (500 and 126 respectively) and internally referenced to the residual solvent signals (^1^H: δ 7.26; ^13^C: δ 77.16 for CDCl_3_, ^1^H: δ 3.31; ^13^C: δ 49.0 for CD_3_OD respectively). NMR chemical shifts (δ) and the coupling constants (J) for ^1^H and ^13^C NMR are reported in parts per million (ppm) and in Hertz, respectively. The following conventions are used for multiplicities: s, singlet; d, doublet; t, triplet; m, multiplet; and dd, doublet of doublet. High resolution mass was recorded on Waters LCT Premier Mass Spectrometer. Absorption spectra were taken on Shimadzu UV-1800 UV-VIS Spectrophotometer. Fluorescence spectra were recorded on Edinburgh FLS980 fluorescence spectrometer and Shimadzu RF-5301pc spectrophotometer.

**Experimental section**

**Fig. S1. Absorbance and emission spectra of the NIR-BG probe before and after activation by β-galactosidase.**

**
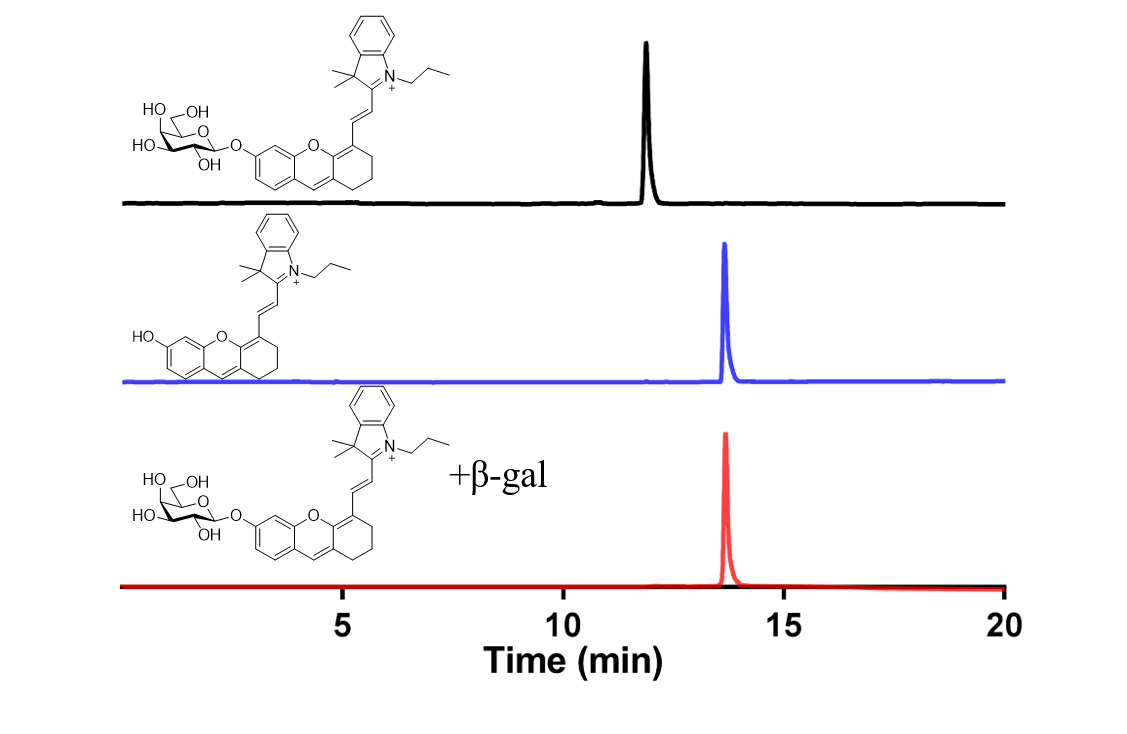
**

Fig. S2. **HPLC traces of NIR-BG probe only (top), standard sample of hydrolyzed end product (middle), NIR-BG probe with β-gal (bottom).**

HPLC analysis of NIR-BG probe activation by **β-galactosidas**. β-Gal (5 unit, 50 μL) was added to the NIR-BG probe solution (30 μL, 1 mM) and the mixture was incubated for 10 minutes at 37 °C. The resulting mixture was then quenched with HCl (1 M, 120 uL). The supernatant was obtained by centrifugation at 10,000 *rpm* for 20 min and then injected into HPLC for analysis. HPLC analysis was performed under the following conditions - mobile phase A: water with 0.1% TFA; B: acetonitrile with 0.1% TFA; 0-10 min: gradient elution, 2-95% B; 10-17min: isocratic elution, 95% B; 17-19 min, gradient elution, 95%-2% B; 19-20 min, isocratic elution, 2%B). The reaction was monitored using UV-vis absorbance at 600 nm.

**Inhibition studies of β-galactosidase.** First, β-gal (0.02 Unit) was incubated for 15 minutes with IPTG (Isopropyl thio-β-D-galactoside) of various concentrations (0.01, 0.05, 0.1, 0.5, 1, 5, 10, 50, 100, 500, 1000, and 2000 μM). Probe (5 μM) was then added to the above solution and the mixture further incubated for 5 min. Fluorescence spectrum was recorded on Shimadzu RF-5301pc spectrophotometer (Setting: Time2DScan, λex = 680 nm, λem = 700 nm, ScanningSpeed = Fast, ExcitationSlitWidth = 5 nm, EmissionSlitWidth = 10 nm, Total Time 5 minutes, AcquisitionRate = 20 Seconds). The fluorescent intensity was plotted as a function of logarithm of inhibitor concentrations.

**Lineweaver-Burke plot of the hydrolysis of NIR-BG probe by β-gal.** The slope of each line was extracted from Fig. 3c. The reaction rate was calculated with these slopes and Fig 2c. Plotting Lineweaver-Burk equation, 1/*V_0_* = *K_M_*/(*K_cat_*[*E*_0_][*S*]) + 1/ (*K_cat_*[*E*_0_]), where [*E_0_*] is the concentration of β-galactosidase , the slope and intercept were obtained from equation: Y = 90.554X+0.8553, R^2^: 0.9996 . The kinetic parameters, *K_M_*, *K_cat_* and *K_cat_*/*K_M,_* were calculated to be 106 μM, 3.3 s^-1^, and 0.31 μM^-1^•s^-1^, respectively.

**Detection Limit.** The detection limit was estimated with the following formula.[[3](#_ENREF_3)]

LOD= 3 σ/s, where σ represents standard deviation of fluorescent intensity of blank sample, s is the calculated slope of linear regression equation. Then LOD was calculated to be 0.0031 unit/mL (0.13 nM) when the value of σ (0.26) and s (255.5) is substituted in above formula.

Synthesis of fluorescence probe

**Fig. S3. Synthetic route to the NIR-BG probe.**

Synthesis of 3. A solution of compound **1** (49.3 mg, 0.12 mmol) and **2** (43 mg, 0.08 mmol) in 3 mL of MeCN was stirred at room temperature. Na_2_SO_4_ (28.4 mg, 0.2 mmol) and Cs_2_CO_3_ (130 mg, 0.4 mmol) were added, and the reaction mixture was stirred at room temperature in the dark for 16 h. Then this mixture was then filtered through a Celite pad and concentrated. The residue was purified by HPLC to afford 3 (25.7 mg, 38%) ^1^H NMR (500 MHz, CDCl_3_) δ 8.68 (d, *J* = 14.5 Hz, 1H), 7.55 – 7.49 (m, 2H), 7.45– 7.36 (m, 3H), 7.18 (s, 1H), 6.98 – 6.96 (m, 2H), 6.48 (d, *J* = 14.5 Hz, 1H), 5.55 – 5.12 (m, 2H), 5.33 – 5.31 (m, 1H), 5.21 – 5.19 (m, 1H), 4.31 – 4.28 (m, 3H), 4.25 – 4.22 (m, 1H), 4.15 – 4.11 (m, 1H), 2.76 – 2.74 (m, 2H), 2.68 – 2.67 (s, 2H), 2.20 (s, 3H), 2.11 (s, 3H), 2.06 (s, 3H), 2.04 (s, 3H),1.98-1.94 (m, 4H), 1.82 (s, 6H), 1.07 (t, *J* = 7.5 Hz, 3H). ^13^C NMR (126 MHz, CDCl_3_) δ 178.21, 170.50, 170.23, 170.08, 169.82, 161.25, 159.29, 153.95, 146.36, 142.08, 141.48, 132.80, 129.39, 128.90, 128.77, 127.80, 122.70, 117.67, 115.15, 113.90, 112.88, 104.58, 98.65, 71.18, 70.82, 68.58, 66.84, 61.09, 51.00, 47.08, 29.82, 29.33, 28.28, 28.18, 24.03, 21.42, 20.89, 20.79, 20.68, 20.25, 11.48. HRMS (ESI) Calcd. For C42H48NO11^+^ [M]^+^: 742.3222; found: 742.3243.

Synthesis of 4. To a solution of 3 (25.7 mg, 0.03 mmol) in 1 mL of MeOH was added 0.7 μL of 25% NaOMe in MeOH. The mixture was stirred at room temperature for 30 min. Then the mixture was purified by HPLC to afford 4 (19.4 mg, 94%). ^1^H NMR (500 MHz, CD_3_OD) δ = 8.81 (d, *J* =15.0 Hz, 1H), 7.68 (d, *J* = 7.5 Hz, 1H), 7.59 – 7.52 (m, 2H), 7.49 – 7.45 (m, 2H), 7.38 (s, 1H), 7.22 (s, 1H), 7.12 (d, *J* = 8.5, 1H), 6.55 (d, *J* = 15.0 Hz, 1H), 5.02 (d, *J* = 7.5 Hz, 1H), 4.35 (t, *J* = 7.0 Hz, 2H), 3.95 (d, *J* = 2.5 Hz, 1H), 3.88 – 3.80 (m, 4H), 3.64 (dd, J=10.0 Hz, 2.0 Hz, 1H), 2.79 (t, *J* = 5.0 Hz, 2H), 2.72 (d, *J* = 6.0 Hz, 2H), 1.98-1.94 (m, 4H), 1.85 (s, 3H), 1.83 (s, 3H), 1.08 (t, *J* = 7.5 Hz, 3H). ^13^C NMR (126 MHz, CD_3_OD) δ = 179.71, 162.78, 162.32, 155.43, 147.39, 143.66, 142.99, 134.51, 130.23, 129.87, 129.26, 128.55, 123.84, 118.27, 116.33, 115.66, 114.09, 105.20, 104.70, 102.99, 77.67, 74.79, 72.07, 70.28, 62.66, 52.17, 47.59, 30.11, 28.41, 28.32, 24.97, 22.31, 21.62, 11.57. HRMS (ESI) Calcd. For C_34_H_40_NO_7_^+^ [M]^+^: 574.2799; found: 574.2803.

**Fig. S4 ^1^H NMR spectrum of intermediate 3 (Fig. S4) in CDCl_3_.**

**Fig. S5 ^13^C NMR spectrum of intermediate 3 (Fig. S4) in CDCl_3_.**


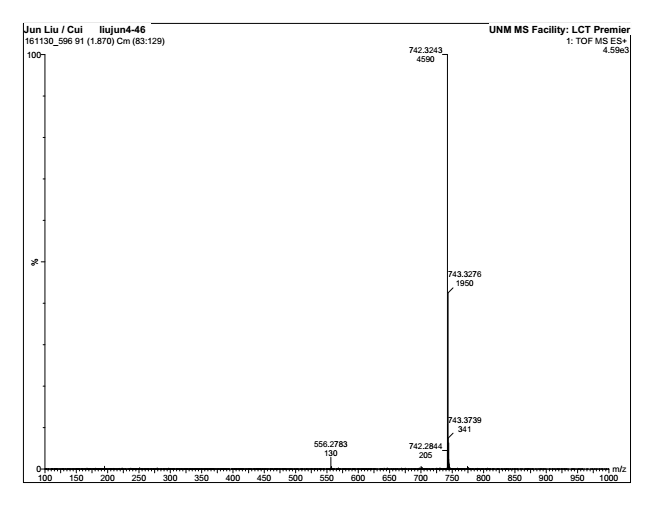


**Fig. S6 ESI-MS spectra of intermediate 3 (Fig. S4).**

**Fig. S7 ^1^H NMR spectrum of the NIR probe in CD_3_OD.**

**Fig. S8 ^13^C NMR spectrum of the NIR probe in CD_3_OD.**

**Fig. S9 ^1^H-^1^H COSY spectrum of the NIR probe in CD_3_OD.**


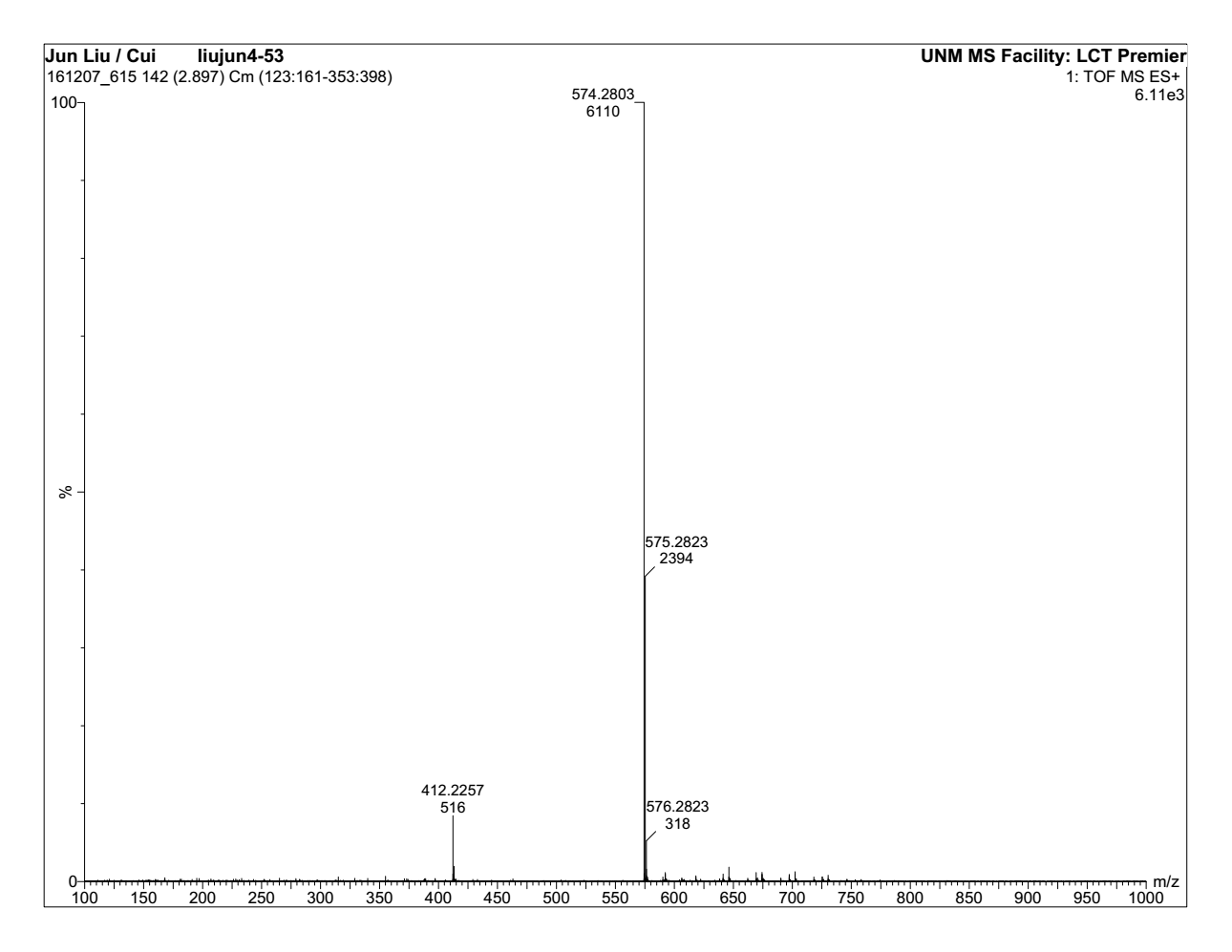


**Fig. S10 ESI-MS spectra of NIR-BG.**

**Stability analysis.** Probe NIR-BG (10 nmol, 100 μL) was added to the black 96-well plate and excited 2s with 640 nm laser for 30 times. The fluorescence intensities after 5, 15, and 30 of laser excitations were compared with the fluorescence intensity of the first excitation. The result showed the fluorescent intensity still higher than 90% after irradiated with laser for 30 times. Except for the laser excitation stability, the stability of the activated probe in enzyme solution was also tested by incubating NIR-BG (10 nmol, 100 μL) with 10 unit of β-gal for different time. The 720 nm fluorescence intensity was measured after 5, 15, 30, and 2880 min of incubation and expressed as percentage of the fluorescence intensity at 5 min. From the Fig. S3 (B), we could observe that the fluorescence intensity still over 80%. These data demonstrating the good stability of NIR-BG, which is good for imaging purpose.


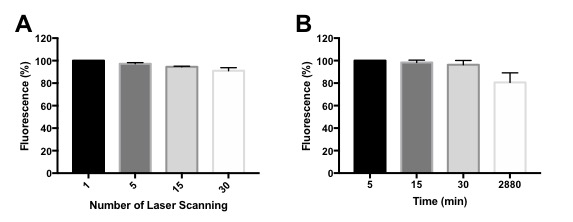


Fig. S11. **Stability assay of probe NIR-BG with laser scanning. A) The fluorescence emission ratio of NIR-BG after different times of laser scanning. B) The fluorescence emission ratio of NIR-BG incubating with β-gal (10 unit) for different time.**

**
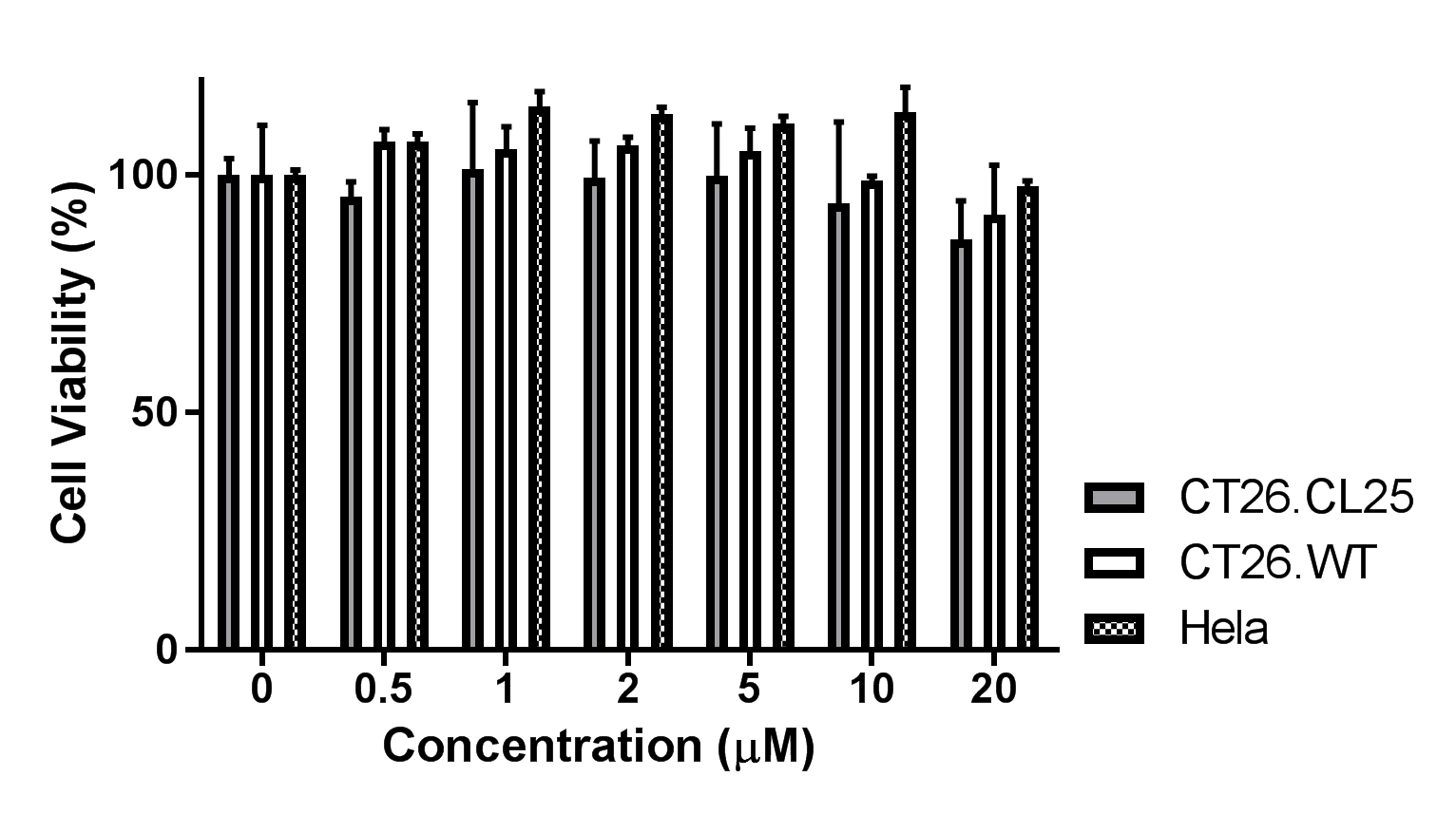
**

**
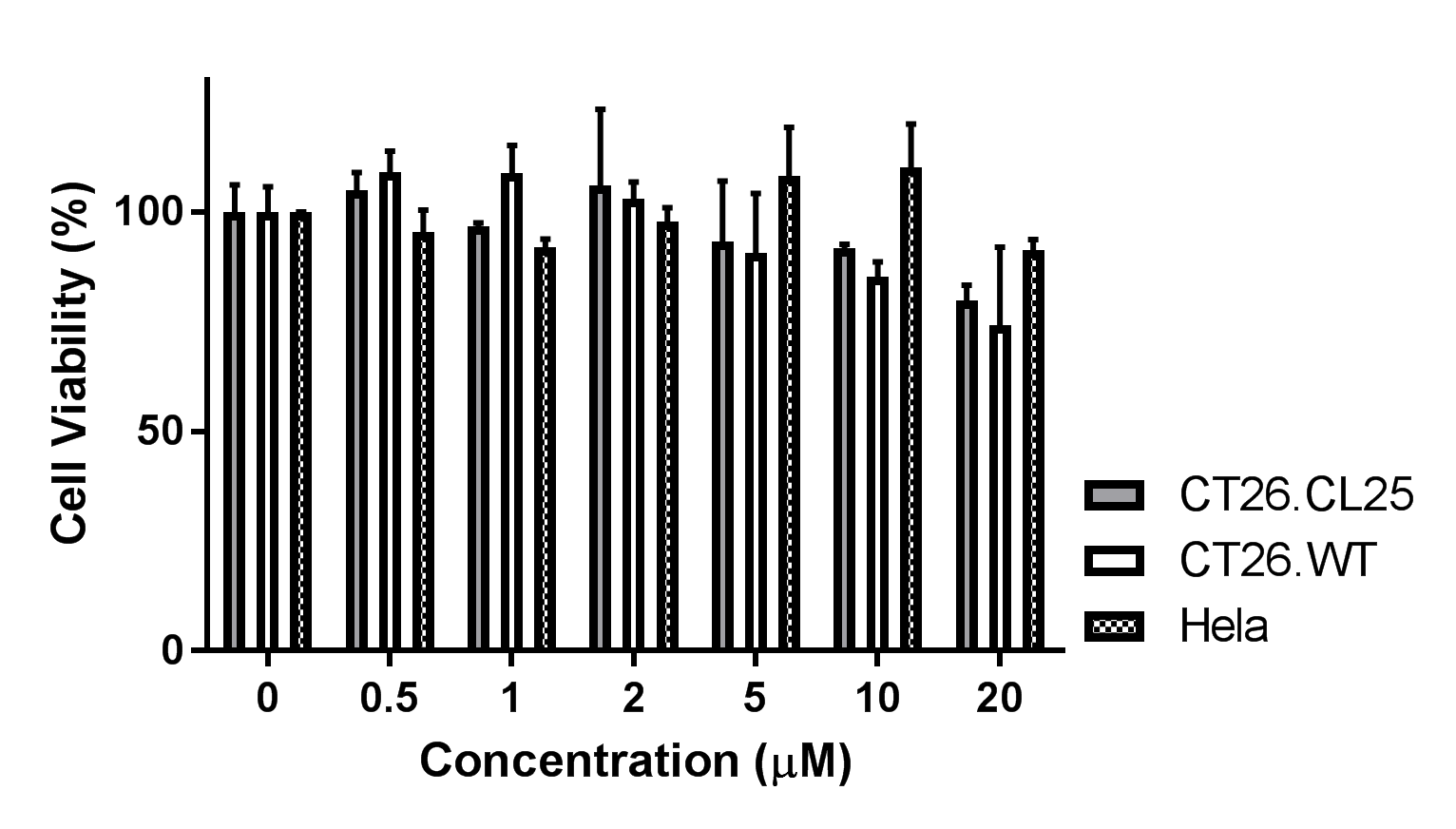
**

Fig. S12. **Cell viability of all the cells in the study after incubation with the NIR-BG probe for 4 hours (top) and 24 hours (bottom).**

**Fig. S13. Immunofluorescent staining of CT26.wt and CT26.CL25 cells. Red indicates probe NIR-BG, green indicates lysosomes (stained with LAMP1 antibody), yellow indicates β-gal.** Note: Anti β-gal antibody was labeled with AF568 (orange fluorescence) for spectral separation during data acquisition, and green pseudocolor is used here for clarity in overlapped images.

**
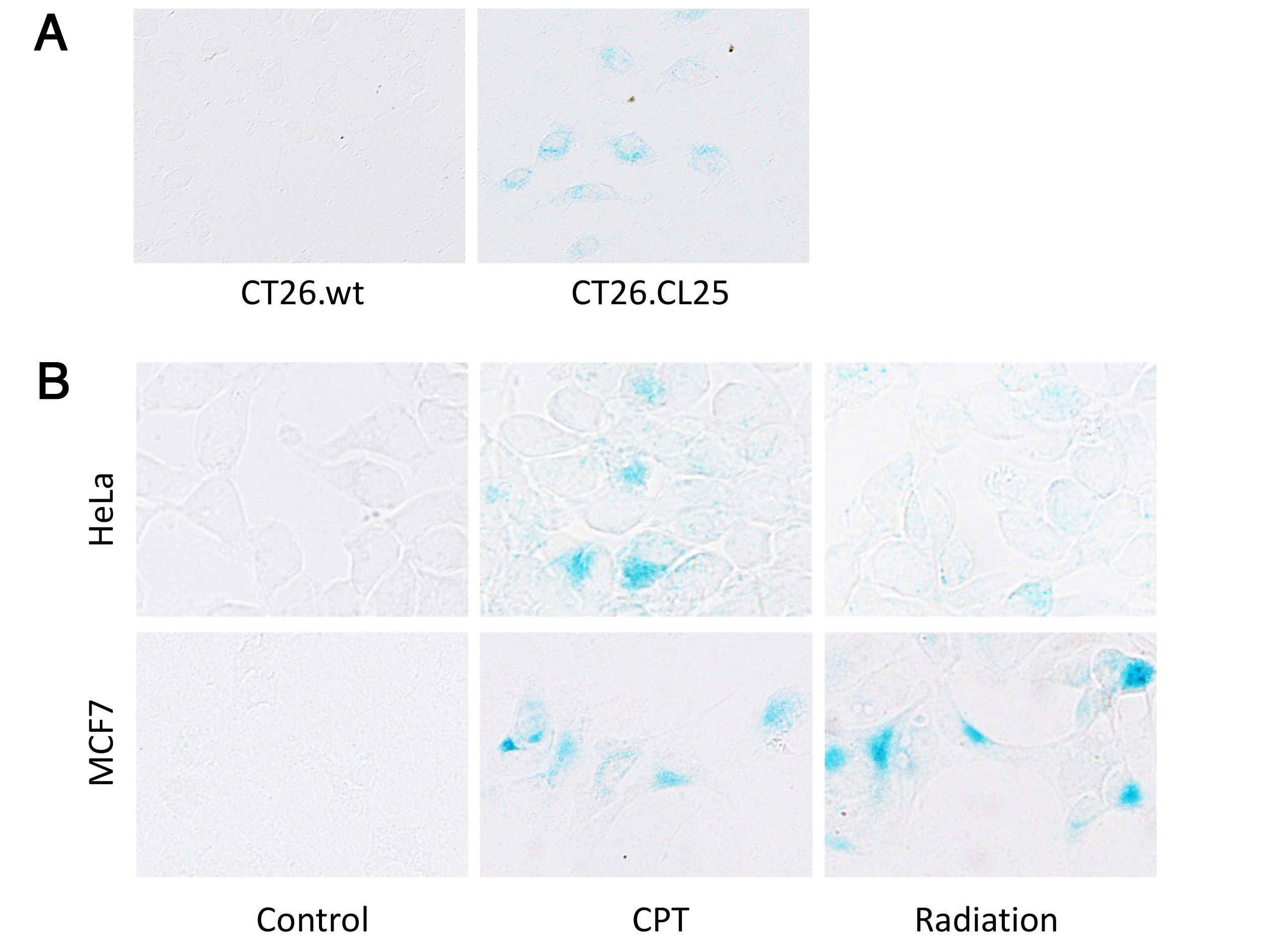
**

Fig. S14. X-gal staining of cells. (A) X-gal staining of CT26.wt and CT26.CL25 cells. (B) X-gal staining of HeLa and MCF7 cells with or without CPT and radiation treatment.

Fig. S15. **SDS-PAGE of lysates of cells treated with CPT (left lane), CPT and CHX (middle lane), or without drug treatment (right lane). Sections A (beta-actin) and B (p16/p21) were used in Western blot analysis.**

**
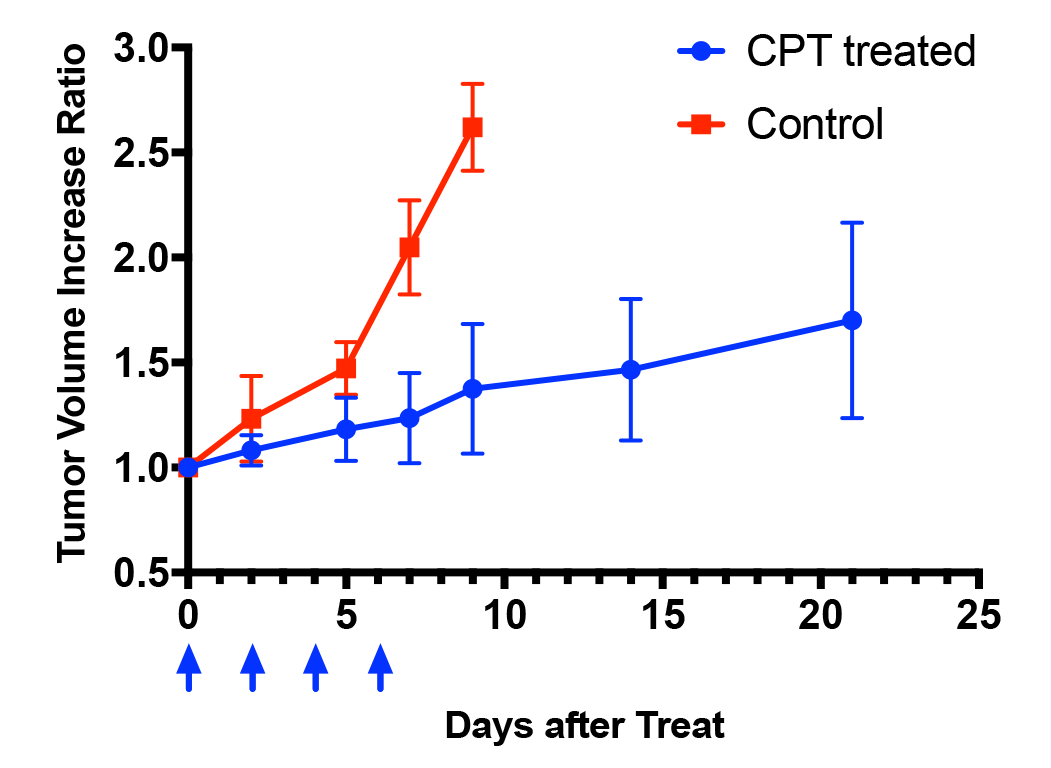
**

Fig. S16. HeLa tumor growth curve with and without CPT treatment. Blue arrows below the x-axis indicate the CPT administration time. (The tumors of control group reached the maximum volume limitation and were imaged and euthanized.)

**
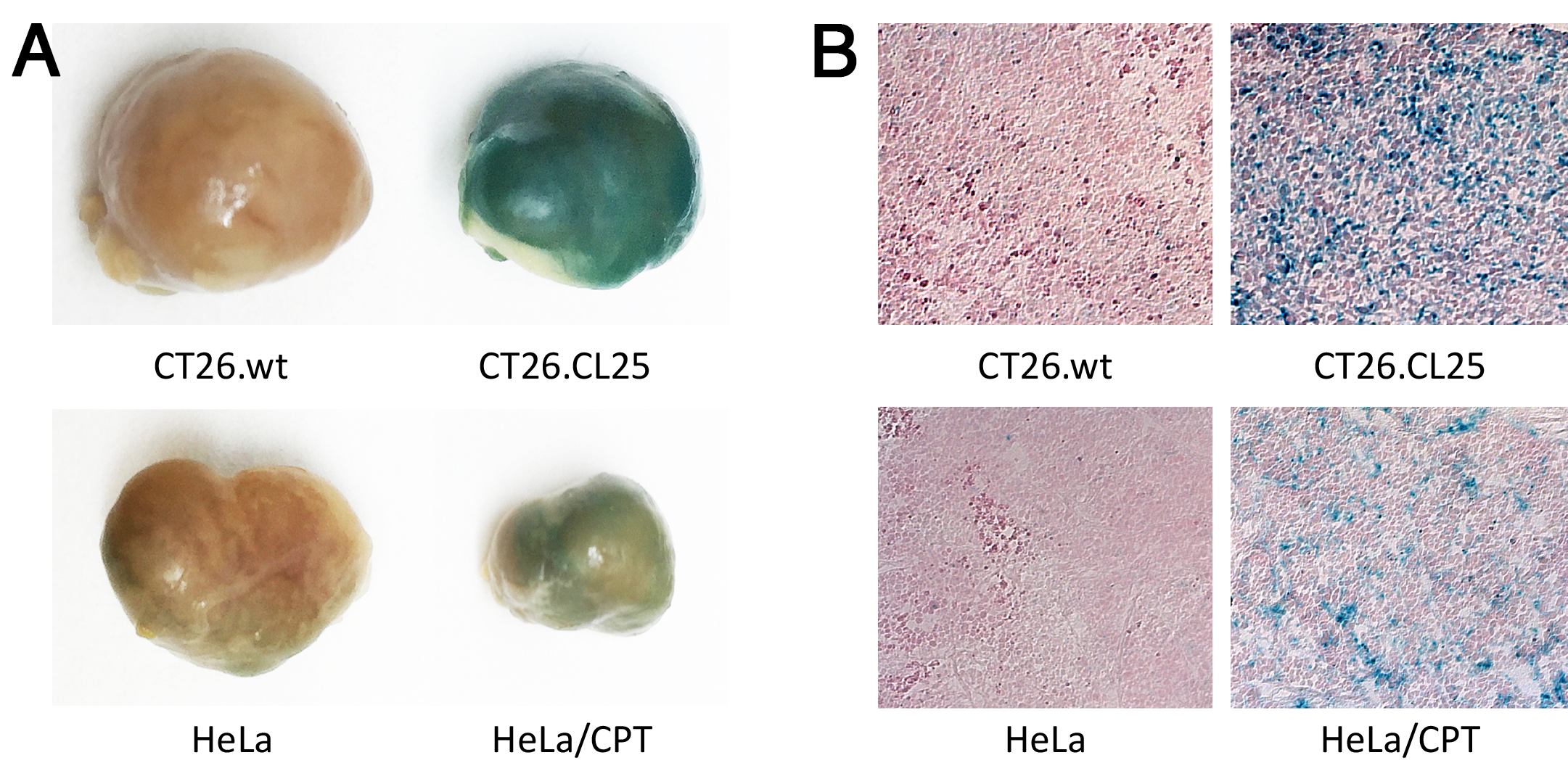
**

Fig. S17. X-gal staining of tumors and tumor slides. (**A) Intact tumor staining with x-gal, blue color indicating the** β-gal expression. **(B) Eosin (pink color) and x-gal (blue color) counter staining of tumor slides. Blue color indicating the over expressed** β-gal **in tumor tissues.**

Fig. S18. **Immunofluorescent staining of tumor tissues. Immunofluorescent imaging of CT26.wt and CT26.CL25 tumor slides. Red indicates NIR-BG probe, green indicates β-gal.** Note: Anti β-gal antibody was labeled with AF568 (orange fluorescence) for spectral separation during data acquisition, and green pseudocolor is used here for clarity in overlapped images.

Fig. S19. **Immunofluorescent imaging of HeLa tumor slides without or with CPT treatment. Red indicates NIR-BG probe, green indicates β-gal.** Note: Anti β-gal antibody was labeled with AF568 (orange fluorescence) for spectral separation during data acquisition, and green pseudocolor is used here for clarity in overlapped images.

**References:**

[1] K.E. Moog, M. Barz, M. Bartneck, F. Beceren-Braun, N. Mohr, Z. Wu, L. Braun, J. Dernedde, E.A. Liehn, F. Tacke, T. Lammers, H. Kunz, R. Zentel, Polymeric Selectin Ligands Mimicking Complex Carbohydrates: From Selectin Binders to Modifiers of Macrophage Migration, Angewandte Chemie, 56 (2017) 1416-1421.

[2] Z. Li, X.Y. He, Z. Wang, R.H. Yang, W. Shi, H.M. Ma, in vivo imaging and detection of nitroreductase in zebrafish by a new near-infrared fluorescence off-on probe, Biosens Bioelectron, 63 (2015) 112-116.

[3] Y. Tan, L. Zhang, K.H. Man, R. Peltier, G. Chen, H. Zhang, L. Zhou, F. Wang, D. Ho, S.Q. Yao, Y. Hu, H. Sun, Reaction-Based Off–On Near-infrared Fluorescent Probe for Imaging Alkaline Phosphatase Activity in Living Cells and Mice, ACS Applied Materials & Interfaces, 9 (2017) 6796-6803.
